# Assessment of Cu, Pb and Zn content in selected species of grasses and in the soil of the roadside embankment

**DOI:** 10.1002/ece3.6627

**Published:** 2020-08-31

**Authors:** Adam Gawryluk, Teresa Wyłupek, Paweł Wolański

**Affiliations:** ^1^ Department of Grassland and Landscape Shaping Faculty of Agrobioengineering University of Life Sciences in Lublin Lublin Poland; ^2^ Department of Agroecology and Landscape Architecture Faculty of Biology and Agriculture Rzeszów University Rzeszów Poland

**Keywords:** bioaccumulation, copper, grasses, heavy metal, lead, phytostabilization, soils, zinc

## Abstract

It was assumed in the study that heavy metals occurring in soils and the air accumulate in grasses constituting the main species used in the turfing of soil in road verges and embankments along traffic routes and in other parts of urbanized areas. The aim of the present study was to assess the bioaccumulation of Cu, Pb, and Zn in three selected lawn cultivars of five grass species and in the soil of the roadside green belt in terms of soil properties and heavy metal uptake by plants in the aspect of determining their usefulness in protecting the soils from contamination caused by motor vehicle traffic. Samples of the plant material and soil were collected for chemical analysis in the autumn of 2018 (October) on the embankment along National Road No. 17 between Piaski and Łopiennik (Poland), where 15 lawn cultivars of five grass species had been sown 2 years earlier. During the study, Cu, Pb, and Zn levels were determined in the aboveground biomass of the grasses under study and in the soil beneath these grasses (the 0–20 cm layer). All the grass species under study can thus be regarded as accumulators of Cu and Zn because the levels of these elements in the aboveground biomass of the grasses were higher than in the soil beneath these grasses. The present study demonstrates that the grasses can accumulate a large amount of Cu and Zn from soils and transfer it to the aboveground biomass. Tested species of grasses are not a higher bioaccumulators for Pb. The best grass species for the sowing of roadsides embankment, with the highest BCF values for the studied metals, is *Lolium perenne* (Taya variety).

## Graphical abstract

The objective of the present study was to identify the relationship between concentrations of heavy metals in roadside soil and the absorption of these metals by grasses. Cu and Zn accumulated the most in *Lolium perenne* while Pb in *Festuca arundinacea*. The analysis of the aboveground biomass of the grasses under study showed a negative correlation between Zn and Pb content in the soil and Cu and Zn content in *Festuca ovina*, and between Pb content in the soil and Pb content in *Poa pratensis*. All the grass species under study can thus be regarded as hyperaccumulators of Cu and Zn because the levels of these elements in the aboveground biomass of the grasses were higher than in the soil beneath these grasses.

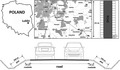

1

Contamination with heavy metals is a global problem and a serious threat to human and animal health all over the world. Some metals in trace amount like Zn or Cu are essential for the growth of plant species. However, increase and excess in its concentration results in detrimental effect like chlorosis, necrosis, toxicity, and stunted growth of plants (Maiti, 2013). Metal accumulation in plants depends on various factors such as type of plant, age, pH, form and type of available metals in substrate and climatic conditions (Maiti & Jaiswal., 2008).

Cu intake by plants depends not only on the species and cultivar, but also on polyamines which increase Cu tolerance in plants (Kováčik, Klejdus, Hedbavny, & Bačkor, 2010; Kováčik et al., 2011). The plants’ intake of Zn from the soil is limited and depends on the pH, the physicochemical properties of the soil, and the activity of microorganisms in the rhizosphere. Zn is absorbed by the roots mostly as a divalent cation (Zn^2+^) (Gupta, Ram, & Kumar, 2016).

The factors which control Zn mobility in the soil are very similar to those listed for Cu, but, unlike other heavy metals, Zn occurs in the soil very frequently in easily soluble forms. The Zn absorption rate differs considerably between plant species. According to Kabata‐Pendias and Pendias (2001), Zn is accumulated the most in the aboveground parts of plants in ecosystems where this pollutant occurs in the air. On the other hand, the solubility and availability of Zn for plants is negatively correlated with the saturation of the soil with Ca and P compounds. According to the investigations by Kabata‐Pendias and Pendias (1999), in the case of a high pH (7.2–7.8), Zn intake by barley is closely correlated with Zn levels in the soil. It is believed that Zn stimulates the plants’ resistance to dry and hot weather as well as bacterial and fungal diseases. On the other hand, the toxicity of Zn depends on the species, genotype, and growth stage. Rout and Das (2003) demonstrated an inhibitory effect of Zn on Cu where the intake of one element inhibited the intake of the other. This may indicate the same mechanisms of the absorption of both metals.

Although Pb is regarded as a metal with the lowest bioavailability to plants, large amounts of it accumulate in plants (mainly the roots). According to Iskandar (2001), the pH of the soil has little influence on the availability of Pb. Other researchers believe that Pb is easily absorbed by plant leaves because stomata leaves are larger than lead particle size (Farid, Shams Farooq, & Khan, 2017). Hamdy, Al Obiady, and Al Mashhady (2015) found Pb occurring in the air and settling on the leaves of plants to be a considerable source of this metal in the aboveground parts of plants. They calculated that up to 95% of the total Pb content in the plants can originate from the Pb suspended in the air. Pb occurring in the soil is bound with carboxylic functional groups of uronic acid contained in the mucus of root cells, or by means of polysaccharides on the surface of the rhizodermis (Seregin & Ivanov, 2001). Studies show that some grass species, such as *Festuca rubra* (Ginn, Szymanowski, & Fein, 2008) or *Paspalum notatum* (Araújo, Lemos, Ferreira, Freitas, & Nogueira, 2007), are able to absorb Pb through their root system. Pb can also be absorbed by the roots by means of passive intake resulting from the difference of concentration levels. It should be remembered, however, that the rhizodermis of the roots prevents Pb from traveling to the aboveground parts of the plants. The biggest amounts of Pb accumulate in the tips of the roots because young root cells have thin cell walls (Hamdy et al., 2015); Seregin, Shpigun, & Ivanov, 2004).

Due to the fact that since 2005 Research Centre For Cultivar Testing in Poland has not been conducting the assessment of the utility value of lawn grass varieties, therefore the sensitivity of these varieties to unfavorable habitat conditions is not known. During this time, there has been a huge progress in breeding lawn grass varieties, both Polish and foreign, which may differ from sensitivity to stressful environmental factors and their suitability for phytoremediation and sodding of contaminated areas.

It was assumed in the study that heavy metals occurring in soils and the air accumulate in grasses constituting the main species used in the turfing of soil in road verges and embankments along traffic routes and in other parts of urbanized areas. The aim of the present study was to assess the bioaccumulation of Cu, Pb, and Zn in three selected lawn cultivars of five grass species and in the soil of the roadside green belt in terms of soil properties and heavy metal uptake by plants in the aspect of determining their usefulness in protecting the soils from contamination caused by motor vehicle traffic.

## 2 MATERIAL AND METHODS

**FIGURE 1:**
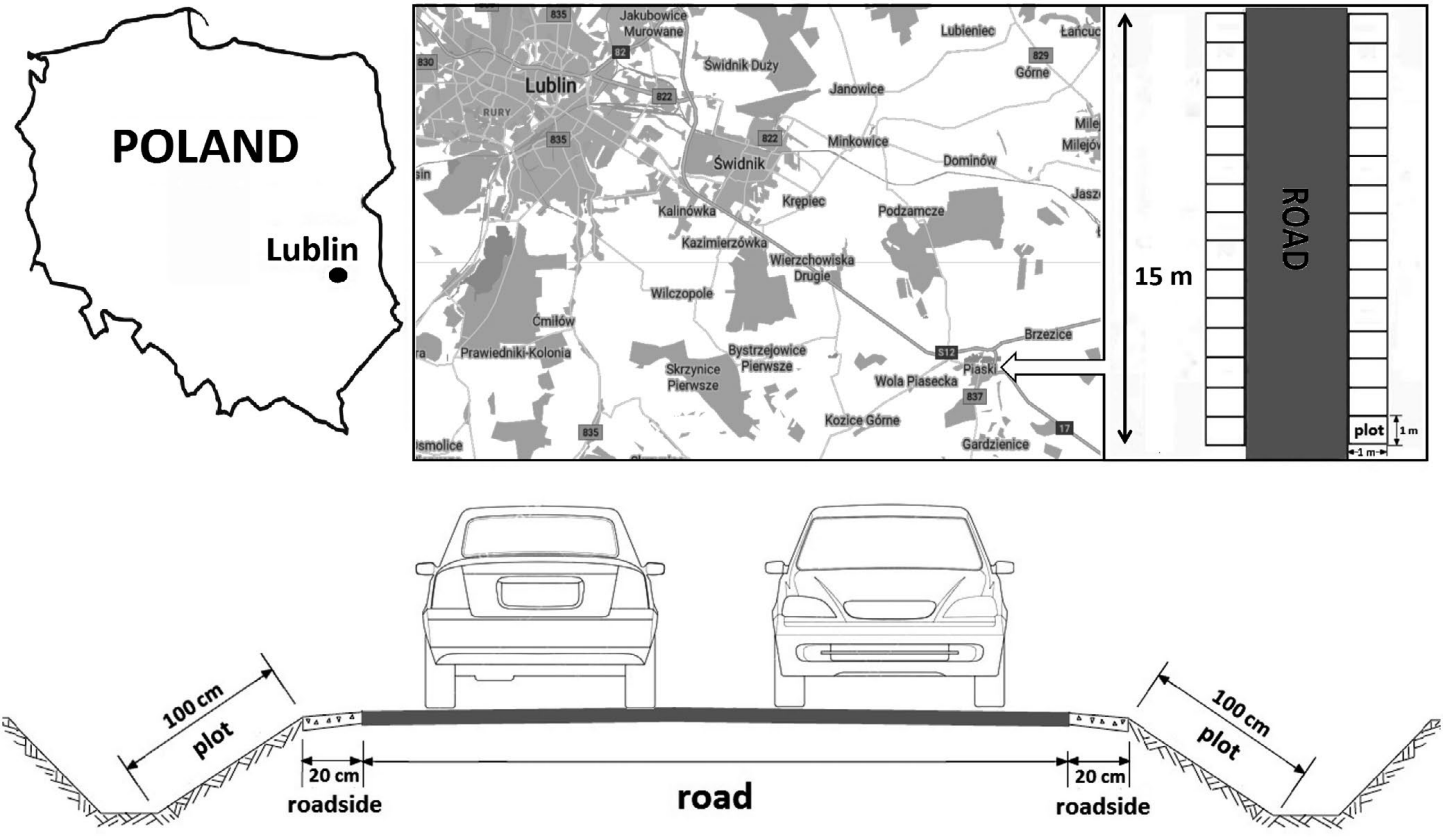
Map of Poland with study area marked

The investigations were conducted in the autumn of 2018 (October) on the embankment along National Road No. 17 between Piaski and Łopiennik (Poland), where 15 lawn cultivars of five grass species had been sown 2 years earlier (October 2016): *Festuca arundinacea* (Asterix, Romina and Tarmena), *Festuca ovina* (Mimi, Tenis and Tomika), *F. rubra* (Areta, Nista and Oliwia), *Lolium perenne* (Natara, Nira and Taya), and *Poa pratensis* (Alicja, Ani and Bila). The experiments were based on the method of randomized blocks in three repetitions. Each variety was sown in monoculture, on microplots covering 1 m^2^ each, according to the methodical recommendations of COBORU (Domański, 1998) and IHAR (Prończuk, Prończuk, & Żyłka, 1997), on both sides of the road (Figure 1). The plots were located about 20 cm from the side of the road. The species selected for investigation are the most frequent and the largest components of grass ecosystems of roadsides (Harkot, Wyłupek, & Czarnecki, 2005; Stawicka, 2003).

The samples of the plant material (aboveground biomass of each species) and soil material were collected for laboratory analysis at about 0.5 m from the edge of the plot, in three repetitions. There were collected nine representative samples of plants and soil (three samples from each plot) per variety, so 27 samples per species (three variety). Soil was sampled from the 0–20 cm layer using a soil probe (Egner's sampler), at the same spots where the plant material was sampled. The soil and plant samples were hidden in hermetic containers (string bags) immediately after collection. Then, the plant samples were washed for 10 s with distilled water, dried, and hidden in airtight containers.

The granulometric composition was determined using the Casagrande areometric method in Prószyński's modification according to PN‐R‐04032:01.12.10. It consists in measuring the density of soil suspension during particle sedimentation using a soil hydrometer, at intervals needed for subsequent fractions to fall. Soil pH was determined using a glass electrode in a soil suspension in a 1 mol/L KCl solution according to PN‐ISO 10390:1997. Soil salinity was measured using a conductometric method according to KQ/PS‐50 ver. 03 from 01.10. 2010. The soil weighed 10 g was shaken in 25 ml distilled water for 1 hr. Then, the conductivity was determined using an Elmetron conductometer. Extraction of P and K was carried out by the Egner–Riehm method, according to PN‐R‐04023:1996 and PN‐R‐04022:1996, respectively. To extract easily soluble forms P and K, a lactate buffer with pH = 3.55 was used. Extraction of absorbable Mg was carried out by the Schachtschabl method in accordance to PN‐R‐04020:1994. Mg was extracted from the soil with an extraction solution (0.0125 M CaCl_2_) at a soil: solution ratio (m/v) of 1:10. The content of P, K, and Mg was determined by the AAS method. In order to prepare samples for the determination of trace elements, plant samples were dried at 70°C, and after drying and grinding, the plants were mineralized in a mixture of HClO_4_ and HNO_3_ (in a 1:4 volume ratio) at 210°C. Soil samples were mineralized in aqua regia (HCl and HNO_3_ in a 3:1 volume ratio) in a microwave oven. Cu, Pb, and Zn levels in the plant and soil material were determined using flame atomic absorption spectroscopy (FAAS)—in accordance with KQ/PB‐16 and KQ/PB‐28 for the plant material, and with KQ/PB‐17 for the soil material. Determination of Cu was performed at a wavelength of 324.8 nm of Zn—213.8 nm and Pb—216.9 nm.

Bioconcentration factor (BCF) of elements in plant material was calculated using the equation:

$$\text{BCF}=\frac{\text{Ps}(x)}{\text{Ss}(x)}$$

BCF—Bioconcentration factor, Ps (*x*)—Content of element *x* in the plant sample, Ss (*x*)—Content of the element *x* in the soil sample (Bech et al., 2012; Wu et al., 2011; Zacchini et al., 2009).

The results of the analysis of heavy metal levels in the plant and soil material were processed statistically in SAS v. 9.1 software by means of variance analysis. Analysis of variance was used to assess differences between means of examined variables for selected grass species and their varieties. Mixed models were used, in which the species (or variety) was a fixed factor, while the plot was used a random factor. Multiple comparison Tukey's test was used to verify the significance of differences between the means assessed. Spearman's rank‐order correlation coefficient was used to determine the correlation between variables. In statistical analysis, significance level *α* = .05 was assumed. The control object consisted of three control plots on each of them three measurements were taken (this is to assess the variability between plots so that the experiment is equally founded).

## 3 RESULTS

In the granulometric composition of the top layer of soil (0–20 cm), fine‐grained gravel predominated (73%), while sand accounted for 9% and fine dust for 8%. The <0.02 mm clay fraction accounted for 10% (Table 1).

**TABLE 1:** Soil grain size composition (% fraction)

Surface layer of the soil (0–20 cm)		
Fractions	Granulometric composition	
	diameter in mm	% of share in fraction
Fine‐grained gravel	2.0–1.0	73
Sand	1.0–0.05	9
Fine dust	0.05–0.02	8
Clay fraction	<0.02	10
	0.02–0.005	6
	0.005–0.002	3
	<0.002	1

The soil samples analyzed had an alkaline pH, and the pH levels did not vary significantly between sites except for soil samples collected from beneath *F. ovina* and *L. perenne*, where the pH was significantly the highest in comparison to the other species and control soil. Salinity was higher in *F. arundinacea*, *F. ovina*, and *F. rubra*, while lower in *L. perenne* and *P. pratensis* compared to control soil. The Mg level was significantly lower in samples of soil from beneath *F. ovina* and *F. rubra*. Compared to control soil, higher P level were found in the soil samples collected from beneath *F. ovina* and *F. rubra*. The higher K level was recorded in samples of soil from beneath *F. arundinacea* compared to control soil (Table 2, Table S1).

**TABLE 2:** Average pH, salinity, selected compounds, and heavy metals in the soil of the examined species of grasses (in mg kg^−1^ d.w.)

Species/variety	pH	Salinity	P	K	Mg	Cu	Zn	Pb
Control	**7.90^a^ ± 0.33**	**0.290^c^ ± 0.02**	**8.46^b^ ± 0.51**	**8.23^bc^ ± 0.29**	**2.40^b^ ± 0.07**	**7.70^b^ ± 0.21**	**42.5^d^ ± 1.14**	**0.9^a^ ± 0.06**
*Festuca arundinacea*	**7.85^a^ ± 0.07**	**0.416^e^ ± 0.01**	**7.88^b^ ± 0.38**	**11.79^d^ ± 0.75**	**2.57^b^ ± 0.13**	**7.81^b^ ± 0.20**	**36.5^b^ ± 0.30**	**20.7^f^ ± 0.51**
Asterix	7.93^y^ ** ± **0.14	0.300^x^ ** ± **0.01	7.48^x^ ** ± **0.66	12.34^y^ ± 1.11	2.70^y^ ** ± **0.18	7.88^y^ ** ± **0.36	39.2^z^ ** ± **0.88	18.2^y^ ** ± **0.47
Romina	7.78^x^ ** ± **0.12	0.411^y^ ** ± **0.02	9.06^y^ ** ± **0.06	13.17^y^ ** ± **0.86	2.70^y^ ** ± **0.18	9.27^z^ ** ± **0.32	36.1^y^ ** ± **0.44	34.8^z^ ± 1.13
Tarmena	7.84^xy^ ** ± **0.11	0.536^z^ ** ± **0.01	7.09^x^ ** ± **0.47	9.85^x^ ± 0.58	2.30^x^ ** ± **0.11	6.29^x^ ** ± **0.38	34.1^x^ ** ± **0.92	9.1^x^ ** ± **0.25
*Festuca rubra*	**7.78^a^ ± 0.09**	**0.330^d^ ± 0.01**	**7.00^a^ ± 0.37**	**7.42^a^ ± 0.31**	**2.06^a^ ± 0.09**	**7.78^b^ ± 0.20**	**39.8^c^ ± 0.75**	**13.7^d^ ± 0.32**
Areta	7.67^x^ ± 0.11	0.311^x^ ** ± **0.01	9.42^z^ ± 0.86	7.82^y^ ** ± **0.74	2.50^y^ ** ± **0.14	8.61^y^ ** ± **0.24	44.3^z^ ** ± **1.12	15.2^z^ ** ± **0.53
Nista	7.67^x^ ± 0.28	0.311^x^ ** ± **0.01	4.98^x^ ** ± **0.053	9.45^z^ ** ± **0.65	2.29^y^ ** ± **0.13	8.07^y^ ** ± **0.27	36.2^x^ ** ± **0.82	12.0^x^ ** ± **0.50
Olivia	8.00^y^ ± 0.17	0.369^y^ ** ± **0.01	6.61^y^ ** ± **0.66	4.98^x^ ** ± **0.20	1.39^x^ ** ± **0.15	6.66^x^ ** ± **0.29	39.0^y^ ** ± **1.05	13.9^y^ ** ± **0.52
*Festuca ovina*	**8.12^b^ ± 0.10**	**0.438^f^ ± 0.01**	**12.77^c^ ± 0.50**	**7.56^ab^ ± 0.46**	**2.11^a^ ± 0.06**	**6.55^a^ ± 0.16**	**39.1^c^ ± 0.77**	**12.0^c^ ± 0.30**
Mimi	8.34^y^ ± 0.17	0.164^x^ ** ± **0.01	7.02^x^ ** ± **0.37	7.82^y^ ** ± **0.74	1.56^x^ ± 0.07	7.09^y^ ** ± **0.19	41.6^y^ ** ± **1.21	13.5^z^ ** ± **0.50
Tenis	8.08^y^ ± 0.17	0.384^y^ ** ± **0.01	24.96^y^ ** ± **0.92	9.30^z^ ** ± **0.82	2.92^z^ ** ± **0.18	6.88^y^ ** ± **0.32	42.6^y^ ** ± **1.23	12.2^y^ ** ± **0.44
Tomika	7.93^x^ ± 0.13	0.764^z^ ** ± **0.02	6.32^x^ ** ± **0.63	5.57^x^ ** ± **0.44	1.86^y^ ** ± **0.07	5.67^x^ ** ± **0.17	33.2^x^ ** ± **0.98	10.3^x^ ** ± **0.31
*Lolium perenne*	**8.09^b^ ± 0.11**	**0.183^a^ ± 0.01**	**7.81^b^ ± 0.37**	**7.56^ab^ ± 0.50**	**2.43^b^ ± 0.04**	**6.77^a^ ± 0.19**	**33.1^a^ ± 0.53**	**10.9^b^ ± 0.20**
Natara	7.74^x^ ** ± **0.19	0.280^y^ ** ± **0.03	4.35^x^ ** ± **0.54	5.57^x^ ** ± **0.44	2.10^y^ ** ± **0.010	9.06^z^ ** ± **0.29	49.1^z^ ** ± **1.28	15.9^z^ ** ± **0.54
Nira	8.35^y^ ** ± **0.16	0.146^x^ ** ± **0.01	7.29^y^ ** ± **0.73	6.16^x^ ** ± **0.74	1.80^x^ ** ± **0.09	7.00^y^ ** ± **0.21	29.3^y^ ** ± **0.89	11.1^y^ ** ± **0.31
Taya	8.18^y^ ** ± **0.22	0.124^x^ ** ± **0.01	11.78^z^ ** ± **0.56	10.96^y^ ** ± **1.12	3.40^z^ ** ± **0.14	4.24^x^ ** ± **0.22	20.9^x^ ** ± **0.78	5.9^x^ ** ± **0.33
*Poa pratensis*	**7.83^a^ ± 0.07**	**0.230^b^ ± 0.01**	**8.21^b^ ± 0.19**	**8.95^c^ ± 0.50**	**2.53^b^ ± 0.17**	**7.90^b^ ± 0.18**	**43.6^d^ ± 0.31**	**15.4^e^ ± 0.19**
Alicja	7.68^x^ ** ± **0.06	0.147^x^ ** ± **0.01	4.95^x^ ** ± **0.59	8.89^y^ ** ± **0.44	2.29^x^ ** ± **0.13	7.78^x^ ** ± **0.43	38.5^x^ ** ± **1.14	18.1^z^ ** ± **0.50
Ani	7.79^x^ ** ± **0.15	0.177^y^ ** ± **0.01	8.39^y^ ** ± **0.35	10.96^z^ ** ± **1.12	3.20^y^ ** ± **0.47	7.76^x^ ** ± **0.032	45.3^y^ ** ± **1.09	13.5^x^ ** ± **0.43
Bila	8.01^x^ ** ± **0.11	0.366^z^ ** ± **0.01	11.29^z^ ** ± **0.59	6.99^x^ ** ± **0.74	2.10^x^ ** ± **0.10	8.16^y^ ** ± **0.26	46.9^z^ ** ± **1.42	14.8^y^ ** ± **0.61

Note

^a–d^—designation of homogeneous species groups at the significance level *α* = .05.

^x,y,z^—designation of groups of homogeneous varieties within the species at the significance level *α* = .05.

Bold ‐ average value for species.

Within the *F. arundinacea* species, the highest salinity level was determined in soil samples from beneath the Tarmena cultivar, and the highest P, K, and Mg levels—in soil samples collected from beneath the Romina cultivar. Within the *F. rubra* species, the highest salinity level was found in soil samples collected from beneath the Olivia cultivar. The highest P and Mg levels were observed in soil samples from beneath the Areta cultivar and the highest K level—in soil samples collected from beneath the Nista cultivar. Within the *F. ovina* species, the highest salinity level was recorded in soil samples from beneath the Tomika cultivar and the highest P, K, and Mg levels—in soil samples collected from beneath the Tenis cultivar. Within the *L. perenne* species, the highest salinity level was found in soil samples from beneath the Natara cultivar, while the highest P, K, and Mg levels—in soil samples from beneath the Taya cultivar. Within the *P. pratensis* species, the highest salinity and P levels were determined in soil samples from beneath the Bila cultivar, and the highest K and Mg levels—in soil samples collected from beneath the Ani cultivar. The lower Cu level was found in samples of soil from beneath *F. ovina* and *L. perenne*, while lower Zn level was determined in samples of soil from beneath all species without *P. pratensis* compared to control soil. The higher Pb level was found in samples of soil from beneath all species compared to control soil (Table 2.)

Within the *F. arundinacea* species, the highest Cu and Pb levels were determined in the soil collected from beneath the Romina cultivar and the highest Zn levels—in the soil from beneath the Asterix cultivar. Within the *F. rubra* species, the highest Cu, Zn, and Pb levels were recorded in the soil from beneath the Areta cultivar. Within the *F. ovina* species, the Cu and Zn levels were the highest in the soil collected from beneath the Mimi and Tenis cultivars, while the highest Pb level was found in the soil from beneath the Mimi cultivar. Within the *L. perenne* species, the highest Cu, Zn, and Pb levels was found in the soil from beneath the Natara cultivar. Among the *P. pratensis* cultivars, the highest Cu and Zn levels was determined in the soil from beneath the Bila cultivar. The highest Pb level was found in the soil from beneath the Alicja cultivar (Table 2).

The analyses showed that the lowest Cu, Zn, and Pb levels (*p* < .05) occurred in the aboveground biomass of *P. pratensis* (Table 3). The highest significant Cu level was found in *L. perenne*, Zn—in *F. rubra,* and Pb—in *F. arundinacea*. Within the *F. arundinacea* species, the Cu, Zn, and Pb levels were recorded in the Romina cultivar. Within the *F. rubra* species, the highest Cu, Zn, and Pb levels were recorded in the leaves of the Nista cultivar. Within the *F. ovina* species, the highest Cu, Zn, and Pb levels were found in the Tomika cultivar. Within the *Lolium Perenne* species, the lowest Cu and Pb levels were found in the Nira cultivar, and the highest Cu levels were found in the Natara cultivar, Zn—in Nira, and Pb—in Taya. Among the cultivars of *P. pratensis*, the highest levels of Cu and Zn were determined in the Bila and Alicja cultivar, and of Pb—in the Ani cultivar (Table 3, Table S2).

**TABLE 3:** The content of Cu, Zn, and Pb in grass of the examined species of grasses (in mg k^−1^ d.w.)

Species/variety	Cu	Zn	Pb
*Festuca arundinacea*	**11.1^b^ ± 0.32**	**57.4^b^ ± 1.15**	**3.55^d^ ± 0.13**
Asterix	10.5^x^ ** ± **0.52	36.3^x^ ** ± **1.54	0.89^x^ ** ± **0.04
Romina	11.7^y^ ** ± **0.46	85.4^z^ ** ± **3.68	8.01^z^ ** ± **0.37
Tarmena	11.2^xy^ ** ± **0.34	50.4^y^ ** ± **2.44	1.75^y^ ** ± **0.10
*Festuca rubra*	**11.5^c^ ± 0.22**	**61.1^d^ ± 0.85**	**2.70^c^ ± 0.08**
Areta	8.6^x^ ** ± **0.39	45.5^x^ ** ± **2.44	1.72^y^ ** ± **0.07
Nista	15.1^z^ ** ± **0.68	87.6^z^ ** ± **3.93	5.31^z^ ** ± **0.26
Olivia	10.9^y^ ** ± **0.44	50.1^y^ ** ± **2.48	1.08^x^ ** ± **0.04
*Festuca ovina*	**12.3^d^ ± 0.23**	**56.7^b^ ± 1.05**	**2.62^c^ ± 0.08**
Mimi	11.6^y^ ** ± **0.46	55.5^y^ ** ± **2.69	2.36^y^ ** ± **0.09
Tenis	9.1^x^ ** ± **0.38	37.8^x^ ** ± **1.95	1.26^x^ ** ± **0.03
Tomika	16.2^zv^ ** ± **0.79	76.7^z^ ** ± **4.17	4.23^z^ ** ± **0.17
*Lolium perenne*	**13.1^e^ ± 0.25**	**59.1^c^ ± 0.94**	**2.10^b^ ± 0.07**
Natara	14.2^z^ ** ± **0.79	47.3^x^ ** ± **1.30	1.78^x^ ** ± **0.09
Nira	12.2^x^ ** ± **0.60	68.0^z^ ** ± **2.98	1.69^x^ ** ± **0.08
Taya	13.0^y^ ** ± **0.56	61.9^y^ ± 2.56	2.82^y^ ± 0.10
*Poa pratensis*	**9.3^a^ ± 0.15**	**44.0^a^ ± 0.29**	**1.50^a^ ± 0.12**
Alicja	9.8^y^ ** ± **0.53	45.3^y^ ** ± **2.28	1.08^x^ ** ± **0.05
Ani	7.8^x^ ** ± **0.20	40.1^x^ ** ± **2.11	2.23^y^ ** ± **0.09
Bila	10.3^y^ ** ± **0.08	46.6^y^ ** ± **1.33	1.18^x^ ** ± **0.04

Note

^a–d^—designation of homogeneous species groups at the significance level *α* = .05.

^x,y,z^—designation of groups of homogeneous varieties within the species at the significance level* α* = .05.

Bold ‐ average value for species.

In all the analyzed soil samples, no significant correlations were found between the soil pH and levels of the heavy metals studied (Table 4). The analysis of control soil samples showed that Cu and Zn levels increased with increasing P level, while Pb level was rising with increasing Mg content. The analysis of soil samples from beneath *F. arundinacea* showed that Zn levels increased with decreasing soil salinity, while Pb level was rising with increasing K content. The analysis of soil samples from beneath *F. rubra* showed that Cu levels in the soil increased with decreasing salinity and Mg levels, while Zn and Pb levels increased with increasing P levels. The analysis of soil samples from beneath *F. ovina* showed that Cu and Pb levels in the soil increased with decreasing soil salinity. On the other hand, in the soil collected from beneath *L. perenne*, increased salinity and decreased P and K levels caused an increase of Cu, Zn, and Pb levels in the soil. In the soil collected from beneath *P. pratensis*, increased salinity and P levels caused an increase of Zn level and decrease Pb level (Table 4).

**TABLE 4:** Relationship between pH value, salinity and content of macroelements and accumulation of heavy metals in soil (for *p* < .05)

Species	Statistic	pH w KCl	Salinity	P	K	Mg
Control						
Cu (Soil)	*r*	0.033	−0.322	0.678	0.267	−0.323
	*p*‐value	0.932	0.398	0.045	0.488	0.397
Zn (Soil)	*r*	−0.133	−0.203	0.803	0.617	0.323
	*p*‐value	0.732	0.600	0.009	0.077	0.397
Pb (Soil)	*r*	−0.042	0.162	0.336	−0.410	0.745
	*p*‐value	0.915	0.678	0.376	0.273	0.021
*Festuca arundinacea*						
Cu (Soil)	*r*	0.101	−0.429	0.718	0.769	0.641
	*p*‐value	0.973	0.126	0.055	0.236	0.098
Zn (Soil)	*r*	0.304	−0.870	0.203	0.567	0.585
	*p*‐value	0.276	<0.001	0.310	0.142	0.202
Pb (Soil)	*r*	−0.048	−0.428	0.745	0.828	0.616
	*p*‐value	0.474	0.124	0.072	<0.001	0.101
*Festuca rubra*						
Cu (Soil)	*r*	0.697	−0.827	0.377	0.465	−0.146
	*p*‐value	0.132	<0.001	0.052	0.214	<0.001
Zn (Soil)	*r*	0.355	−0.517	0.892	−0.399	0.332
	*p*‐value	0.818	0.801	<0.001	0.139	0.137
Pb (Soil)	*r*	0.705	−0.906	0.876	−0.419	−0.308
	*p*‐value	0.886	0.985	<0.001	0.080	0.159
*Festuca ovina*						
Cu (Soil)	*r*	−0.084	−0.843	0.482	0.575	0.843
	*p*‐value	0.068	<0.001	0.111	0.062	0.279
Zn (Soil)	*r*	0.465	−0.423	0.730	0.763	0.304
	*p*‐value	0.052	0.065	0.070	0.095	0.163
Pb (Soil)	*r*	0.465	−0.432	0.310	0.456	0.281
	*p*‐value	0.075	<0.001	0.116	0.077	0.059
*Lolium perenne*						
Cu (Soil)	*r*	−0.533	0.840	−0.893	−0.774	−0.455
	*p*‐value	0.134	<0.001	<0.001	<0.001	0.057
Zn (Soil)	*r*	−0.536	0.830	−0.880	−0.798	−0.460
	*p*‐value	0.154	<0.001	<0.001	<0.001	0.065
Pb (Soil)	*r*	−0.547	0.858	−0.843	−0.746	−0.431
	*p*‐value	0.073	<0.001	<0.001	<0.001	0.125
*Poa pratensis*						
Cu (Soil)	*r*	0.577	0.315	0.327	−0.448	−0.556
	*p*‐value	0.082	0.148	0.096	0.089	0.076
Zn (Soil)	*r*	0.653	0.811	0.822	−0.301	−0.162
	*p*‐value	0.055	<0.001	<0.001	0.127	0.473
Pb (Soil)	*r*	−0.306	−0.415	−0.448	−0.304	−0.498
	*p*‐value	0.096	0.032	0.019	0.124	0.088

In all the analyzed samples of the plant material, no significant correlations were found between the soil pH and salinity on the one hand and heavy metal levels on the other (Table 5). The analysis of the aboveground parts of *F. ovina* showed that Cu and Zn levels in the leaves were rising with decreasing P level, and Pb level in the leaves increased with decreasing K levels. A relationship was found in *F. rubra* between an increase in P levels in the soil and decreased Zn and Pb levels in the plants as well as between increased K levels in the soil and increased Cu, Pb, and Zn levels in the aboveground parts of *F. rubra*. Analyses also showed that Pb levels in the aboveground parts of *L. perenne* increased with increasing Mg levels in the soil. In the case of the other species, no significant relationships (no correlations) were found between macro‐element levels and the accumulation of heavy metals in the plants (Table 5).

**TABLE 5:** Relationship between pH value, salinity and content of macroelements and accumulation of heavy metals in grass species (for *p* < .05)

Species	Statistic	pH w KCl	Salinity	P	K	Mg
*Festuca arundinacea*						
Cu (Grass)	*r*	−0.345	0.380	0.452	0.135	−0.059
	*p*‐value	0.061	0.141	0.118	0.502	0.636
Zn (Grass)	*r*	−0.480	0.455	0.621	0.244	−0.031
	*p*‐value	0.106	0.216	0.201	0.220	0.882
Pb (Grass)	*r*	−0.390	0.439	0.590	0.182	0.002
	p‐value	0.223	0.071	0.351	0.364	0.999
*Festuca rubra*						
Cu (Grass)	*r*	−0.317	0.415	0.802	0.820	−0.497
	*p*‐value	0.971	0.840	0.065	0.030	0.100
Zn (Grass)	*r*	−0.293	0.456	−0.898	0.419	−0.493
	*p*‐value	0.998	0.967	<0.001	0.022	0.101
Pb (Grass)	*r*	−0.277	0.481	−0.870	0.440	−0.514
	*p*‐value	0.084	0.063	0.020	<0.001	0.108
*Festuca ovina*						
Cu (Grass)	*r*	−0.419	0.433	−0.446	0.891	−0.326
	*p*‐value	0.098	0.132	<0.001	0.087	0.221
Zn (Grass)	*r*	−0.538	0.459	−0.458	0.920	−0.319
	*p*‐value	0.144	0.218	<0.001	0.096	0.117
Pb (Grass)	*r*	−0.436	−0.445	−0.793	−0.829	0.500
	*p*‐value	0.167	0.071	0.136	<0.001	0.318
*Lolium perenne*						
Cu (Grass)	*r*	−0.728	0.511	−0.855	−0.869	0.394
	*p*‐value	0.253	0.165	0.123	0.181	0.149
Zn (Grass)	*r*	0.799	−0.483	−0.873	−0.889	−0.443
	*p*‐value	0.075	0.401	0.064	0.328	0.202
Pb (Grass)	*r*	−0.132	−0.386	−0.858	−0.915	0.835
	*p*‐value	0.559	0.056	0.084	0.111	<0.001
*Poa pratensis*						
Cu (Grass)	*r*	0.429	0.314	−0.435	−0.266	−0.738
	*p*‐value	0.238	0.123	0.119	0.056	0.086
Zn (Grass)	*r*	0.405	0.326	0.410	0.196	−0.755
	*p*‐value	0.248	0.122	0.135	0.102	0.074
Pb (Grass)	*r*	0.150	0.384	0.540	0.624	0.571
	*p*‐value	0.346	0.089	0.078	0.315	0.131

Spearman's rank‐order correlation analysis showed that Cu and Zn levels in the aboveground parts of *F. ovina* were rising with decreasing Zn and Pb levels in the soil. In *P. pratensis*, on the other hand, Pb levels increased in the aboveground parts with decreasing Pb levels in the soil. In the case of the other species, no significant relationships were found between heavy metal levels and their accumulation in the plants (Table 6).

**TABLE 6:** Relationship between heavy metal content in soil and heavy metal accumulation in grass species(for *p* < .05)

Species	Statistic	Cu (Soil)	Zn (Soil)	Pb (Soil)
*Festuca arundinacea*				
Cu (Grass)	*r*	0.287	−0.261	0.277
	*p*‐value	0.226	0.167	0.253
Zn (Grass)	*r*	0.414	−0.441	0.454
	*p*‐value	0.229	0.120	0.216
Pb (Grass)	*r*	0 0.427	−0.482	0 0.466
	*p*‐value	0.326	0.110	0.313
*Festuca rubra*				
Cu (Grass)	*r*	−0.517	−0.764	−0.452
	*p*‐value	0.080	0.063	0.052
Zn (Grass)	*r*	−0.551	−0.776	−0.437
	*p*‐value	0.071	0.057	0.067
Pb (Grass)	*r*	−0.605	−0.736	−0.466
	*p*‐value	0.133	0.124	0.133
*Festuca ovina*				
Cu (Grass)	*r*	−0.349	−0.891	−0.951
	*p*‐value	0.105	<0.001	0.020
Zn (Grass)	*r*	−0.362	−0.890	−0.877
	*p*‐value	0.212	<0.001	0.022
Pb (Grass)	*r*	0.412	−0.432	−0.419
	*p*‐value	0.331	0.056	0.085
*Lolium perenne*				
Cu (Grass)	*r*	0.404	0.453	0.433
	*p*‐value	0.138	0.116	0.122
Zn (Grass)	*r*	−0.475	−0.430	−0.450
	*p*‐value	0.054	0.066	0.120
Pb (Grass)	*r*	−0.526	−0.464	−0.507
	*p*‐value	0.065	0.215	0.056
*Poa pratensis*				
Cu (Grass)	*r*	0 0.450	0.155	0.425
	*p*‐value	0.082	0.469	0.129
Zn (Grass)	*r*	0.368	0.196	0.431
	*p*‐value	0.102	0.373	0.225
Pb (Grass)	*r*	−0.114	0.478	−0.825
	*p*‐value	0.509	0.212	<0.001

Assessing the degree of bioconcentration of heavy metals, the highest BCF for the Pb, Zn, and Cu was calculated for *L. perenne* and *F. ovina*, and the lowest for *P. pratensis*. The tested species showed a high BCF for Cu—above 1. While BCF for Zn was higher than 1 in most of the tested varieties except Asterix (*F. arundinacea*), Tenis (*F. ovina*), Natara (*Lolium prerenne*), Ani and Bila (*P. pratensis*). The lowest BCF was calculated for Pb, around 0.2 (Table 7).

**TABLE 7:** Bioconcentration factor (BCF) heavy metal on species and varieties of grass

Species/variety	Cu	Zn	Pb
*Festuca arundinacea*	**1.42 ± 0.06**	**1.57 ± 0.04**	**0.17 ± 0.01**
Asterix	1.33** ± **0.11	0.92** ± **0.01	0.05** ± **0.01
Romina	1.26** ± **0.07	2.35** ± **0.07	0.23** ± **0.01
Tarmena	1.78** ± **0.13	1.48** ± **0.07	0.19** ± **0.01
*Festuca rubra*	**1.49 ± 0.06**	**1.53 ± 0.03**	**0.20 ± 0.01**
Areta	1.01** ± **0.04	1.02** ± **0.05	0.11** ± **0.01
Nista	1.86** ± **0.09	2.41** ± **0.05	0.44** ± **0.02
Olivia	1.64** ± **0.10	1.28** ± **0.06	0.08** ± **0.01
*Festuca ovina*	**1.88 ± 0.05**	**1.45 ± 0.01**	**0.22 ± 0.01**
Mimi	1.64** ± **0.08	1.34** ± **0.02	0.17** ± **0.02
Tenis	1.31** ± **0.06	0.89** ± **0.04	0.10** ± **0.01
Tomika	2.86** ± **0.05	2.32** ± **0.09	0.41** ± **0.02
*Lolium perenne*	**1.94 ± 0.08**	**1.78 ± 0.02**	**0.19 ± 0.01**
Natara	1.57** ± **0.07	0.96** ± **0.02	0.11** ± **0.01
Nira	1.73** ± **0.10	2.32** ± **0.05	0.15** ± **0.01
Taya	3.07** ± **0.21	2.96** ± **0.16	0.11** ± **0.02
*Poa pratensis*	**1.18 ± 0.04**	**1.01 ± 0.01**	**0.10 ± 0.01**
Alicja	1.26** ± **0.08	1.18** ± **0.05	0.06** ± **0.02
Ani	1.01** ± **0.01	0.89** ± **0.03	0.17** ± **0.01
Bila	1.26** ± **0.06	0.99** ± **0.03	0.08** ± **0.01

Note

Bold ‐ average value for species.

## 4 DISCUSSION

The investigations showed that fine‐grained gravel predominated (73%), while sand accounted for 9% and fine dust for 8% in the granulometric composition of the top layer of soil (0–20 cm). Thus, soil water deficit can occur in years with small precipitation volumes. This is because the size of granulometric fractions is closely correlated with the physicochemical properties of the soil such as hygroscopicity and coefficient of wilting which reach their maximum levels in the colloidal clay fraction (Paluszek, 2011).

The studied soils had low salinity levels within the natural range of NaCl levels in soil (Huliszet, Charzyński, & Giani, 2010). The analyzed soil samples were alkaline, and the soil pH levels were different between plots. A higher pH of the soils may have been caused by the alkaline character of street dust (Kusińska, Bauman‐Kaszubska, & Dzięgielewska‐Sitko, 2005; Łabuda, 2005; Walczak & Chutko, 2014) that was settling on the roadside embankment. Literature data indicate that in soils with a neutral or alkaline pH, it is harder for metals to penetrate into the deeper layers of the soil profile (Chojnacka, Chojnacki, Gorecka, & Górecki, 2005). A high pH results immobilize metals in the soil through the formation of carbonates and phosphates, among others (Dzierżanowski & Gawroński, 2011).

Our investigations showed that Cu, Zn, and Pb levels in the analyzed soil samples did not exceed the natural levels of these elements in soils in Poland (Chojnacka et al., 2005; Kabata‐Pendias & Pendias, 2001). Cu content in the tested soil samples ranged from 4.24 to 9.27 mg/kg (Table 2); therefore, it did not exceed the natural content of this element in Polish soils (2–20 mg/kg) (Kabata‐Pendias & Pendias, 2001). The Pb content in the studied soils ranged from 9.1 to 20.7 mg/kg (Table 3) and did not exceed the permissible value (30 mg/kg) specified in the Regulation of the Minister of the Environment (2002). Also, the zinc content in the tested soil samples did not exceed the permissible value (300 mg/kg) specified in the Regulation of the Ministry of Environment (2002). On the other hand, the mean heavy metal levels in the grasses were higher than in the soils collected from beneath these grasses. This may have been caused by the settling of pollutants from the air on the leaves and their assimilation by the plants (Chojnacka et al., 2005; Yan et al., 2012). In their studies of heavy metal content in grasses Onder, Dursun, Gezgin, and Demirbas (2007) also found that Cu and Zn levels in the grasses were higher than in the soil. On the other hand, Amusan, Bada, and Salami (2009) and Wei, Ge, Chu, and Feng (2015) found that heavy metal levels in the grasses were considerably lower than in the soil. Also in the study by Onder et al. (2007), Pb levels in the aboveground parts of grasses were lower than in the soil. The levels of heavy metals in the analyzed grasses can be arranged in the following order: Zn > Cu > Pb. The same order of the accumulation of heavy metals by grasses was found in studies by Dinelli and Lombini (1996), Puschenreiter and Horak (2000), and Boularbah et al. (2006).

The metal accumulation efficiency in plants can be evaluated using the bioconcentration factor (BCF), which is defined as the ratio of metal concentration in the plant biomass to metal concentration in the soil. According to Netty, Wardiyati, Maghfoer, and Handayanto (2013) BCF value of 1–10 indicates hyperaccumulator plant, BCF values of 0.1–1 indicate moderate accumulator plant, BCF value of 0.01–0.1 indicates low accumulator plant, and BCF value of <0.01 indicates non‐accumulator plant. Also Zhang, Cai, Tu, and Ma (2002) defined hyperaccumulating plants as those which have a BCF higher than 1. The term hyperaccumulator was first used by Brooks, Lee, Reeves, and Jaffre (1977) and was originally used to define plants containing more than 1,000 ng/g(ppm) nickel in dry tissue. Kramer (2010) stated in his review that hyperaccumulation concentration criterion for Cu and Pb are >1,000 µg/g, while for Zn > 10,000 µg/g. Our research has shown that all tested grass species were good accumulators of Cu, while moderate accumulators for Pb and Zn. In turn, Kumar, Ahirwal, Maiti, and Das (2015) found that *Saccharum munja* and *Cynodon dactylon* were good Pb and Zn hyperaccumulators.

Based on studies by Gupta et al. (2009), and Cenkci et al. (2010), we know about the interactions between Pb and Zn. Lead has a strong affinity with sulfhydryl groups (‐SH) occurring in proteins forming part of metalloid enzymes, as a result of which these enzymes do not bind microelements such as Fe or Zn (Gupta et al., 2009). The possibility of easy availability and movement of metals between root and shoot is usually determined by correlating the metal concentration in different plant parts (Pandey, Singh, Singh, & Singh, 2012). To assess the negative and positive translocation and allocation of metals, correlation coefficient is an important statistical tool to determine the phytoavailability of different metals in soils. Soil chemical composition and the plant species are the two most important factors which determine the phytoavailability of heavy metals (Kumar et al., 2015). Our study did not reveal any significant correlations between the soil pH and levels of the heavy metals studied. This is consistent with the previously published data that does not show any correlation between the soil pH and levels of heavy metals in soil (Conesa, Faz, & Arnaldos, 2006). Puschenreiter and Horak (2000) also found that the soil pH has a small influence on Zn and Cu levels in soil. The analyses of soil samples showed a positive correlation between NaCl levels in the soil and increased Cu, Zn, and Pb levels in the soil from beneath *L. perenne*, while in the other species (except for *P. pratensis*), a decrease in NaCl levels caused an increase in the levels of the studied metals in the soil. The investigation of the soil samples showed an inhibitory effect of P level on Cu, Zn, and Pb levels in the soil from beneath *L. perenne,* and on Zn levels in the soil from beneath *F. ovina* and *P. pratensis*. A decrease of P level in the soils caused an increase in the levels of Zn and Pb in the aboveground parts of *F. rubra*, and of Cu and Zn in the above ground parts of *F. ovina*. The same interactions between the primary elements and trace elements in plants are confirmed in the studies by Kabata‐Pendias and Pendias (2001). Studies also showed an inhibitory effect of K in the soil on Cu, Zn, and Pb levels in the aboveground parts of *F. rubra* as well as a synergic effect of K levels in the soil on Pb levels in the aboveground parts of *F. ovina*, and of Mg levels on Pb levels in the aboveground parts of *L. perenne*.

High levels of inorganic phosphates (Pi) in the soil increase the adsorption of Zn into soil particles (Perez‐Novo, Bermudez‐Couso, Lopez‐Periago, Fernandez‐ Calvino, & Arias‐Estevez, 2011). P levels have a significant impact on the soil pH, which, in turn, influences the mobility of Zn in the soil. Phosphates, along with Zn, may form the insoluble Zn_3_(PO_4_)_2_ that prevents the intake of Zn by the roots of plants. This was confirmed in studies by Huang, Barker, Langridge, Smith, and Graham (2000) and Zhu, Smith, and Smith (2001) who demonstrated an excessive accumulation of P in the shoots of cereals during a deficiency of Zn in the soil.

Cu, Zn, and Pb levels in the aboveground biomass of the grasses did not exceed the permissible levels of these elements in plants (Boularbah et al., 2006; Kabata‐Pendias & Pendias, 2001). According to Kramer (2010), critical toxicity level for Cu is 20–30 μg/g, for Pb—0.6–28 μg/g, and for Zn—100–300 μg/g. The investigations did not find any significant correlations between Cu levels in the soils and Cu, Zn, and Pb levels in the grasses under study. Madejón, Murillo, Maraňón, Cabrera, and López (2002) did not find any significant correlation between Cu levels in the soil and in the grasses either. Our investigations showed a negative correlation between Zn levels in the soil and Cu and Zn levels in *F. ovina* and between Pb levels in the soil and Cu Zn levels in the aboveground parts of *F. ovina* and Pb levels in the aboveground parts of *P. pratensis*. This may result from the fact that Cu levels in the soil are significantly correlated with Zn and Pb levels while there is no correlation between Cu and Cd (Zhang et al., 2012). Kumar et al. (2015) noted positive significant correlation between Zn concentration in soil and Zn concentration in shoot of *S. munja*, as well as a positive correlation between Pb content in soil and aerial parts of *C. dactylon*. Other studies indicate that Cu and Zn levels in plants are influenced by the total Zn content in the soil. These studies showed that high Cu and Zn levels in plants occur in soils with low Zn levels (Puschenreiter & Horak, 2000). On the other hand, studies by Madejón et al. (2002) found a significantly positive correlation between Zn and Pb levels in soil and their levels in grasses. Studies by Abratowska (2006) also indicated a high capacity of Zn to travel from the soil to the aboveground parts of the rye under study.

It should be stressed that the bioavailability of Pb for plants can change under the influence of changes in pH, levels of organic compounds and iron oxides, and amount of phosphorus. Pb intake by the plants is inhibited by elements such as Ca, S, and P which cause the precipitation of Pb in poorly soluble forms (Kabata‐Pendias & Pendias, 2001). Zn is absorbed by plants most frequently in proportion to its levels in the soil even though the properties of the soil and the choice of species have a significant impact on its accumulation in plants (Kabata‐Pendias & Mukherjee, 2007; Kabata‐Pendias & Pendias, 2001).

## 5 CONCLUSIONS

All the grass species under study can thus be regarded as accumulators of Cu and Zn because the levels of these elements in the aboveground biomass of the grasses were higher than in the soil beneath these grasses. The present study demonstrates that the grasses can accumulate a large amount of Cu and Zn from soils and transfer it to the aboveground biomass. Tested species of grasses are not a higher bioaccumulators for Pb. The best grass species for the sowing of roadsides embankment, with the highest BCF values for the studied metals, is *L. perenne* (Taya variety).

## CONFLICT OF INTEREST

The authors declare that they have no conflict of interest.

## AUTHOR CONTRIBUTIONS

**Adam Gawryluk:** Conceptualization (equal); data curation (equal); formal analysis (equal); funding acquisition (equal); investigation (equal); methodology (equal); writing – original draft (lead); writing – review & editing (lead). **Teresa Wyłupek:** Data curation (equal); investigation (equal). **Paweł Wolański:** Investigation (equal); software (equal); writing – original draft (supporting).

## Supporting information

Table S1‐S2

## Data Availability

All data are available in the manuscript.
