# Supplementary material for: Assessment of Cu, Pb and Zn content in selected species of grasses and in the soil of the roadside embankment
Source: Ecol Evol. 2020 Aug 31;10(18):9841–52. doi: 10.1002/ece3.6627 (PMC7520191; doi:10.1002/ece3.6627)
Supplement: Supplementary file 1 — Table S1‐S2 [file ECE3-10-9841-s001.doc]

Table S1. ANOVA results for content and metal accumulation in soil

|  | **pH** | **Salinity** | **P** | **K** | **Mg** | **Cu** | **Zn** | **Pb** |
| --- | --- | --- | --- | --- | --- | --- | --- | --- |
| ***P*- value** |  |  |  |  |  |  |  |  |
| **Species** | <0.001 | <0.001 | <0.001 | <0.001 | <0.001 | <0.001 | <0.001 | <0.001 |
| **Variety** |  |  |  |  |  |  |  |  |
| *Festuca arundinacea* | 0.504 | <0.001 | <0.001 | <0.001 | <0.001 | <0.001 | <0.001 | <0.001 |
| *Festuca rubra* | <0.001 | <0.001 | <0.001 | <0.001 | <0.001 | <0.001 | <0.001 | <0.001 |
| *Festuca ovina* | <0.001 | <0.001 | <0.001 | <0.001 | <0.001 | <0.001 | <0.001 | <0.001 |
| *Lolium perenne* | <0.001 | <0.001 | <0.001 | <0.001 | <0.001 | <0.001 | <0.001 | <0.001 |
| *Poa pratensis* | <0.001 | <0.001 | <0.001 | <0.001 | <0.001 | <0.001 | <0.001 | <0.001 |

Table S2. ANOVA results for content and metal accumulation in plants

|  | **Cu** | **Zn** | **Pb** |
| --- | --- | --- | --- |
| ***P*- value** |  |  |  |
| **Species** | <0.001 | <0.001 | <0.001 |
| **Variety** |  |  |  |
| *Festuca arundinacea* | <0.001 | <0.001 | <0.001 |
| *Festuca rubra* | <0.001 | <0.001 | <0.001 |
| *Festuca ovina* | <0.001 | <0.001 | <0.001 |
| *Lolium perenne* | <0.001 | <0.001 | <0.001 |
| *Poa pratensis* | <0.001 | <0.001 | <0.001 |
